# Use of Over-The-Counter Analgesics Is Associated with Pain, Stress, and Quality of Life in Norwegian Adolescents: A Cross-Sectional Study

**DOI:** 10.3390/children11101247

**Published:** 2024-10-16

**Authors:** Siv Skarstein, Sølvi Helseth, Milada Cvancarova, Kristin Haraldstad, Gudrun Rohde, Hilde Timenes Mikkelsen, Erik Grasaas

**Affiliations:** 1 Department of Nursing and Health Promotion, Faculty of Health Sciences, Oslo Metropolitan University, 0130 Oslo, Norway; 2 Department of Health and Nursing, Faculty of Health and Sport Sciences, University of Agder, 4630 Kristiansand, Norwaygudrun.e.rohde@uia.no (G.R.); hilde.e.mikkelsen@uia.no (H.T.M.); 3 Department of Clinical Research, Sorlandet Hospital, 4615 Kristiansand, Norway; 4 Department of Nutrition and Public Health, Faculty of Health and Sport Sciences, University of Agder, 4630 Kristiansand, Norway; erik.grasaas@uia.no

**Keywords:** children, adolescents, over-the-counter analgesics, OTCA, pain, stress, health-related quality of life (HRQOL)

## Abstract

**Introduction**: Approximately 20% of Norwegian adolescents are frequently using Over-the-Counter Analgesics (OTCAs). The WHO emphasizes the need for research to identify the key determinants of health problems in adolescence. Thus, our aim was to describe and explore pain, stress, and Health-Related Quality of Life (HRQOL) in Norwegian adolescents and investigate possible associations upon high/low usage of OTCAs. **Methods**: This cross-sectional study included 315 adolescents (92 boys, 223 girls) with an average age of 14.1 years (13–15 years). All participants reported using OTCAs. Weekly usage was categorized as high and less than weekly as low. Using a validated questionnaire, we explored the following variables: pain, as measured using the Brief Pain Inventory, stress (Perceived Stress Questionnaire), and HRQOL (KIDSSCREEN-27). Binary logistic regression models were conducted using IBM SPSS Statistics (version 27). **Results**: Our descriptive findings reveal that high users of OTCAs reported higher pain intensity of 3.4 (SD = 1.9) and perceived stress of 0.38 (SD = 0.18) compared to low users, who reported 2.5 (SD = 1.9) and 0.32 (SD = 0.16), respectively. High OTCA users reported lower average scores than low users across all HRQOL subscales. Binary logistic regression revealed 30% higher odds for higher levels of pain intensity and 14 times higher odds of perceived stress associated with being a high user of OTCAs compared to a low user. **Conclusions**: Our study shows significantly higher odds for experiencing pain and stress among adolescents using OTCAs daily-to-weekly, compared to those seldom using such medicines. Health professionals should be aware of young people who have a high consumption of OTCAs and investigate whether the use is related to pain or stress. This might be important in designing a personalized and appropriate intervention. Parents and caregivers have an important responsibility in supporting adolescents’ appropriate pain management. Longitudinal studies are needed to better explore predictive factors of OTCA use in adolescents, particularly in relation to psychological variables such as stress and quality of life.

## 1. Introduction

Over-the-Counter Analgesics (OTCAs), also named non-prescript analgesics, are pain medicines legally available without a prescription from health professionals. The most-selling OTCAs are paracetamol (acetaminophen) and non-steroidal anti-inflammatory drugs (NSAIDs) [1,2]. The sale of OTCAs in Norway is regulated by legislation and regulations, and the medicines can be purchased in pharmacies, supermarkets, and petrol stations [3]. Norwegian sales of both prescription pain medication and OTCAs have increased markedly during the last decade [4]. Paracetamol accounted for more than half of the Norwegian sales of OTCAs in 2020 and is one of the most popular and most used analgesics around the world [3,5]. Mostly, these medicines are available without a prescription, both in mono- and multi-component preparations [5]. In Europe, paracetamol is the most used OTCA, and health authorities recommend paracetamol as the first choice to treat mild to moderate pain [6]. Used as recommended, OTCAs are considered relatively harmless [6]. Also, in other countries, frequent use of OTCAs is rather common among adolescents [7,8]. However, OTCAs may have possible negative side effects and be a threat to health when used in high doses, outside the indications for use, or over a longer duration [5,9].

Adolescence is a vulnerable stage in life [10]. The age group of 13–15-year-old adolescents is particularly significant in terms of developmental transitions, both physically and emotionally. It is a time when many young people begin to make independent choices about health and lifestyle, making it a critical period for studying behavioral patterns [11,12]. Adolescents utilize a diverse range of medications and approaches to self-treat their pain starting from the age of eleven years [13]. Studies show that more girls than boys report a frequent intake of OTCAs [7,8,14,15], and even though most adolescents maintain a responsible attitude toward the utilization of OTCAs, some exhibit a more careless approach [16]. Parents’ attitude towards pain management and OTCAs influences both adolescents’ perception of pain and their use of OTCAs [17]. In general, puberty and adolescence are characterized by increased stress levels, academic pressure, and social expectations and can be experienced as stressful [10]. Further, stress over time might reduce the pain threshold [18]. During the second year of the COVID-19 pandemic, a significant deterioration in the mental health of adolescents was observed, alongside a marked increase in incidents of self-poisoning with paracetamol [15]. This trend raises alarming concerns about the profound impact of the pandemic on young people’s mental health and the consequential risky behaviors they may engage in as a result. The containment measures implemented in response to the COVID-19 pandemic, including quarantine procedures, lockdown protocols, and school closures, drastically altered the daily lives of adolescents. These changes resulted in prolonged periods of isolation, disruptions to normal routines, and loss of contact with social and support networks. Consequently, the lack of access to regular social interactions and support systems that schools provide increased feelings of loneliness and distress among adolescents. Uncertainty and fear surrounding the virus itself, combined with the inundation of news and information about its spread and impact, may have contributed to heightened anxiety levels in this population. Such a state of stress may exacerbate pre-existing mental health issues or trigger the onset of new ones, leading to a state of desperation that might provoke self-harm behaviors as a coping mechanism [19].

One main indication for use of OTCAs is pain [20]. According to the International Association for the Study of Pain, pain is always a personal experience that is influenced by biological, psychological, and social factors [21]. Pain in adolescents has increased during the last two decades and is recognized as a substantial public health challenge in industrialized countries [22]. Among adolescents, pain is the primary reason for using OTCAs. Specifically, headaches are a dominant factor driving the use of OTCA [23]. Adolescents suffering from pain are found to have higher levels of distress, anxiety, sleep disturbance, and lower mood than their peers, which could potentially put them at risk of entering adulthood with mental and physical problems [24]. Pain and stress are found to be prevalent in adolescents, and there appear to be significant stress–pain associations across genders [25]. Pain and use of OTCAs are also associated with self-esteem, depression, and reduced Health-Related Quality of Life (HRQOL) [26].

HRQOL is a multidimensional construct that includes the individual’s subjective perspectives on the physical, psychological, social, and functional aspects of health [27]. The World Health Organization (WHO) emphasizes HRQOL as a goal for public health, especially among adolescents, and underlines the need for research to identify the key determinants for health problems in this age group [28]. HRQOL in adolescents is negatively affected by chronic pain and stress [29,30]. Stress can be considered as a cognitive transaction of the individual’s perception of situations within the psychosocial environment, based upon the outcome of a “demands-coping capacity” appraisal [31].

There is scarce research evidence regarding the influence of pain, stress, and HRQOL on adolescents’ use of OTCAs. Based on the literature presented, there seems to be a possibility that these variables may be related to adolescents’ consumption of OTCAs. Therefore, this study aimed to describe pain, stress, and HRQOL in Norwegian adolescents 13–15 years of age and investigate possible associations between these variables and high/low use of OTCAs.

## 2. Materials and Methods

The present cross-sectional study is a part of the “Start Young—quality-of-life and pain in generations” study, which is a longitudinal study that aims to acquire new knowledge about HRQOL and pain in adolescents and their parents, as well as investigate potential family and regional patterns [32]. The Start Young study was conducted in the south-eastern part of Norway, with approximately 1.6 million inhabitants (30% of the total Norwegian population) and an adolescent population (aged 14–15 years) of approximately 37,000.

The present study included Norwegian adolescents aged 13 to 15 years who had used OTCAs within the last four weeks. Schools covering 9th grade in elementary school were stratified according to region, rural and urban districts, and school size. Two schools were randomized from each stratum. The data collection took place between November 2018 and April 2019.

### 2.1. Participants

The sample consisted of 315 adolescents who, according to self-report, had used OTCAs during the last four weeks. The Start Young study included 696 adolescents in 9th grade from 22 schools in Norway, of which 381 adolescents did not report use of OTCAs within the last four weeks and were not included.

### 2.2. Data Collection

Approximately one week before the data collection took place, project members visited each school and gave the adolescents verbal and written information. Written information was also distributed to the parents. Participants were included if they provided signed consent from both the adolescent and one parent. A web-based questionnaire was administrated and completed at school. One or two project members and a teacher were present in class to offer support when needed. The collected data were stored and analyzed in a secure environment (services for sensitive data (TSD), as approved by the Norwegian Centre for Research Data (NSD: Ref: 60981).

### 2.3. The Questionnaire

The first part of the questionnaire included self-reported data on demographic details such as gender, age, cohabitant status, parental marital status, if parents were born in Norway or abroad, whether the respondent had moved during the previous 5 years, and frequency of school absence. The second part included data on HRQOL, pain, stress, and use of OTCAs, see Table 1.

### 2.4. Use of OTCA

Selected questions derived from the Norwegian “Pain, youth and self-medication study” were used to measure the intake of OTCAs [23]. The respondents were asked about OTCA intake during the last 4 weeks. Further, the respondents were asked about the frequency of intake. The response categories were (1) daily, (2) every week but not daily, and (3) less often than weekly. In our analysis, we defined daily-to-weekly use of OTCAs as high users, whereas those using OTCAs less than weekly were defined as low users. This classification was made by the research group, which consisted of experienced health personnel with considerable experience working with young people. The group assessed the indications and recommendation in the package leaflet stating that it was considered inappropriate consumption if young people without a diagnosed illness or injury used OTCAs daily-to-weekly over four consecutive weeks. The terms “high and low users of OTCA” were also used in an earlier study [23] and have shown relevance when applied to adolescents. According to Skarstein (2014), high users in this age group may not fully comprehend the risks of OTCAs, thereby necessitating targeted interventions [23].

### 2.5. Pain

Pain was assessed using one selected question from the Brief Pain Inventory (BPI), which asks participants to rate the subjective intensity of pain on average. The item is presented as a numeric rating scale, from 0  =  no pain to 10  =  pain as bad as you can imagine. The Norwegian BPI has satisfactory psychometric properties and has been used among both adolescents and adults [33].

### 2.6. Stress

Stress was measured by the Perceived Stress Questionnaire (PSQ) [34]. This is a 30-item questionnaire referring to the last 4 weeks, further answered with a four-point scale with a range from 1 (“almost never”) to 4 (“almost always”). In our analyses, the answers were recoded so that higher values always indicated higher levels of PSQ. The resulting PSQ total score is linearly transformed between zero and one; PSQ = (raw value − 30)/90. Commonly used cut-off levels of stress with respect to the PSQ are low <0.33; medium 0.33 < 0.45; moderate 0.45 < 0.60; and severe 0.60. The Norwegian version of this instrument has been shown to have both good reliability and validity [35].

### 2.7. Health-Related Quality of Life (HRQOL)

HRQOL was measured with the Norwegian version of the KIDSCREEN-27 questionnaire. The KIDSCREEN-27 is a validated, multidimensional measure of generic HRQOL in children and adolescents organized into the following five subscales: physical well-being, psychological well-being, autonomy and parent relations, social support and peers, and school environment. A five-point Likert scale indicates either the frequency of certain behaviors or feelings (ranging from “never” to “always”) or the intensity of an attitude (ranging from “not at all” to “extremely”) within the last week. Rasch-scores were added for subscales and transformed to t-values. These values are normed to a mean of 50 and a standard deviation of 10. Answers were recoded and higher values indicate better HRQOL. The Norwegian version of the instrument has been shown as reliable and valid elsewhere [36].

The assessment tool intakes of OTCA, BPI, PSQ, and KIDSCREEN 27 were chosen for this study due to their proven reliability and validity in evaluating the variables of interest. The intake of OTCA was selected because it allows for a direct and straightforward measure of OTCA use, also used in earlier studies [19,23]. The BPI [30] is widely recognized for its effectiveness in assessing pain severity and the impact of pain on daily functions, making it a valuable tool for our study. The Norwegian version of PSQ [31,32] was chosen to provide a comprehensive understanding of the individual’s pain sensitivity, which can greatly influence their use of pain medications. Lastly, the KIDSCREEN 27, a HRQOL assessment for children and adolescents [33], was selected because of its robustness in capturing the overall well-being of the participants, which is a crucial aspect of our study. These tools were preferred over others due to their relevance to our research questions, their established reliability, and their wide acceptance in similar studies. Their use not only strengthens our experimental design but also enhances the persuasiveness and validity of our results.

### 2.8. Statistical Analyses

All statistical analyses were conducted using IBM SPSS Statistics for Windows, Version 29 (IBM Corp., Armonk, NY, USA). Continuous variables were described using mean and standard deviations (SDs), and categorical variables are presented with counts and percentages. Possible associations between the dependent variables “high user group” versus “low user group” and selected predicting psychosocial independent variables were modelled using binary regression. The final models were adjusted for gender, ethnicity, and living conditions. The results were presented as odds ratios (ORs), with 95% confidence intervals. Missing values were present for <5% of all questions; thus, no imputation of missing values was considered necessary. *p*-values < 0.05 were considered statistically significant, and all tests were two-sided. As the study is considered exploratory, no correction for multiple testing was performed.

## 3. Results

In this study, a total of 315 Norwegian adolescents, 223 girls (60.8%) and 92 boys (29.2%), reported use of OTCAs during the last 4 weeks. Seventy-two adolescents (20%) reported using OTCAs daily or at least weekly (defined here as high users), whereas the remaining 80% of our sample (*n* = 243) used OTCAs less than weekly (low users). Proportions of high users were similar for both genders, as 20 out of 92 boys (21.7%) and 52 of 171 of the girls (22.9%) were categorized as high users.

### 3.1. Descriptive Results for Pain, Stress, and HRQOL

Descriptive findings reveal better outcomes among low users of OTCAs than in the high users of OTCA (see Table 2), with an average pain score of 2.5 (SD, 1.9) versus 3.4 (SD, 1.9), respectively. Moreover, the highest reported findings of stress and lowest reported findings in all HRQOL subscales were revealed in the high OTCA user group.

### 3.2. Associations between Pain, Stress, and HRQOL in Low and High OTCA Users

Stress and pain were strong and independent predictive factors for being a high user of OTCAs (*p* < 0.05). Crude associations revealed an OR of 1.29 for pain intensity (95% CI [1.10; 1.50]) in the high users of OTCAs, compared to the low users of OTCAs (listed in Table 3). Our analysis also revealed 14.7 times higher odds (95% CI [1.08; 200.1]) of stress in the high users of OTCAs compared to the low users of OTCA, which remained significant after adjusting for sociodemographic variables. Regressions revealed nonsignificant findings in four out of five HRQOL subscales, but 8% higher odds of psychological well-being were revealed in the high users of OTCAs compared to the low users of OTCAs after controlling for covariates (95% CI [1.02; 1.15]). See Table 3.

## 4. Discussion

In this current study, we aimed to describe pain, stress, and HRQOL in Norwegian adolescents stratified by high/low use of OTCAs and investigate possible associations between these variables upon high/low use of OTCAs. Descriptive findings reveal that high users of OTCAs had higher pain intensity, higher perceived stress, and lower HRQOL in all domains compared with low users of OTCAs. Binary logistic regression revealed higher odds for pain, perceived stress, and psychological well-being in high users of OTCAs compared to low users.

Our results demonstrate a distinct trend of poorer outcomes for the study variables of pain and stress in the high OTCA user group compared to the low user group. In the high user group, the odds of experiencing pain were highly increased. Further, the one most notable observation derived from our analysis is the statistically significant odds ratio of 14.5, indicating an augmented risk of stress in the high OTCA group. Being low or high users of OTCAs did not appear to affect four out of five HRQOL subscales.

Our study revealed an unexpected finding: there was an 8% higher likelihood of increased psychological well-being in the high OTCA user group compared to the low OTCA user group. This result contradicts our initial expectations and is not consistent with previous studies. In the existing literature, high usage of OTCA has been generally associated with a greater prevalence of pain and psychological problems [17,19,23]. Several studies have suggested a direct correlation between high OTCA use and the emergence or exacerbation of psychological conditions such as anxiety and depression [19]. This belief assumes that frequent use of OTCA may be indicative of attempts to manage untreated or undiagnosed psychological issues. However, our findings provide a contrasting perspective. The high OTCA user group in our study demonstrated an 8% higher probability of improved psychological well-being. This could suggest that these individuals may be effectively using OTCA to manage their psychological conditions, resulting in a higher level of psychological well-being. Alternatively, this could also indicate that the high OTCA user group in our study may represent a different demographic or population compared to those in previous studies, hence the differing results. Another possible explanation could be the role of factors that we did not account for in our research. For instance, the high OTCA user group might have had better access to mental health resources, or they might have been more proactive in seeking help and using other coping strategies, which could have contributed to their increased psychological well-being. Despite these potential explanations, our findings should be interpreted with caution. More research is necessary to fully understand the relationship between high OTCA use and psychological well-being. Future studies could benefit from a more detailed examination of the characteristics of high OTCA users and whether there are specific circumstances or factors that may affect the relationship between OTCA use and psychological health. While our findings challenge the traditional understanding of the relationship between OTCA use and psychological well-being, they also open new avenues for further research into this complex and nuanced issue. However, this may also be due to a combination of low sample size and imprecise estimates, combined with conditions other than psychological well-being that are more relevant to predicting a high consumption of OTCAs. Finally, in this study, sociodemographic factors and gender appear to exert a relatively minor influence on the adolescents’ use of OTCAs, and as such, they did not impact the associations as covariates.

Our findings show that those using OTCAs to a high extent have more stress; this is in line with studies suggesting that some adolescents may resort to OTCAs as a coping mechanism to deal with emotional or psychological stressors [37]. Hence, it is important to acknowledge the possibility of bi-directional associations between stress and OTCA use. Adolescents with a high perception of stress might use OTCAs as a type of pain management. Pain can be a stressor, and stress may lower the pain threshold [38].

The adolescents in the high user group have 30% increased risk for subjective intensity of pain on average. Also, previous studies show similar association between use of OTCAs and pain [23,37]. Not surprisingly, the high user group has higher odds of increased pain intensity (OR 3.4) than the low user group (OR 2.5). Even if the difference is significant, however, there is a large proportion of young people in the low consumer group who have pain but seldom use OTCAs. In the future, it would be of interest to study possible differences in pain-related coping strategies, resilience, and health literacy. However, the occurrence of pain is also high in the low user group. According to King, chronic pain in adolescents affects 20–25% of the youth population [39]. Research evidence has shown that chronic pain is associated with decreased HRQOL and significant psychosocial (e.g., anxiety, depression) as well as pain-related impairment (e.g., decreased physical activity, use of assistive devices) [29]. This highlights the complexity regarding pain and stress in adolescents. Research also indicates that depression and traumatic experiences might contribute to the usage of OTCAs [37]. However, it is also important to be aware of learned coping strategies and the influence of parents’ attitudes towards use of OTCAs [17].

In our study, the proportions of boys and girls with high use of OTCAs are nearly identical. However, it is important to note that the study lacked measures that could provide further insight into earlier findings that have shown that adolescent females appear to report more significant interpersonal stress than males and that the relationship between major life stress and psychological symptoms seems stronger for adolescent females than males [40].

The high prevalence of OTCA use among children is worrying because adolescence is a critical stage of development when lifestyle habits are developed. Behavioral patterns learned in childhood are likely to progress into adulthood [41]. Most adolescents appeared to have responsible attitudes toward use of OTCAs, even if some use them more carelessly [16]. It might be that some adolescents turn to OTCAs to cope with emotional or psychological distress, using pain relief to temporarily alleviate their feelings [26]. Others may seldom use OTCAs even though they have pain. Differences in learned pain management and parents’ attitude toward OTCAs seem to be possible explanations [17].

Adolescents are highly susceptible to peer influence and may be motivated to imitate their friends or peers [42]. This can create a perception that it is a common or socially acceptable practice to use OTCAs frequently. Studies also show that adolescents may not be fully aware of the appropriate dosage and frequency of use for over-the-counter medications [43,44]. Further, lack of knowledge about proper use and side effects may lead them to mistakenly believe that taking more medication than recommended will provide better or faster relief [44]. It might also be that adolescents in the high user group lack alternative coping strategies to manage pain and stress. For example, they may not have learned or developed healthier coping mechanisms, such as exercise, relaxation techniques, or talking to a trusted adult. High use of OTCAs for coping with pain and stress may prevent adolescents from learning healthier coping mechanisms.

### 4.1. Implications and Contribution

The pandemic’s influence on high school students’ interest in healthcare careers, especially among females and freshmen, can help address projected professional shortages. Policies should encourage healthcare support, enhance mental health support, tailor career guidance, enrich the curriculum, promote equity, and address stereotypes for a resilient workforce. It is important for adolescents to learn to cope with pain and stress in a way that promotes HRQOL. Therefore, targeted research is needed to investigate how adolescents with a high consumption of OTCAs can be supported, as this would be most useful for health professionals, such as school nurses and General Practitioners.

### 4.2. Strengths and Limitations

The overall response rate for the Start Young study at baseline was only 41.8%, thus indicating a relatively high level of non-participation. Unfortunately, we do not have information to assess whether the participants and nonparticipants differed in any respect [32]. A further limitation is the self-selection of the participants, which may have influenced the representativeness of the sample. In addition, the relatively low participation rate could indicate a bias. This could potentially affect the accuracy of the data, leading to skewed results and conclusions. There are significant gender differences in the incidence of pain and OTCA use in adolescents; however, our sample collection did not fully consider gender differences, which may lead to biased results. Given the nature of this cross-sectional study, it is essential to acknowledge that the establishment of directional relationships and causal connections is not assured; therefore, delving into longitudinal study designs would likely provide more comprehensive insights. Moreover, as our high user of OTCAs sample was relatively small, highly unprecise estimates were identified in the confident intervals of the regressions analysis for perceived stress, which reduced the validity of findings and should be considered a limitation. The survey utilizes a broad interpretation of OTCAs, as none of the survey questions explicitly inquire about the specific OTCA types. The definition employed for OTCAs does not consider prescriptions from healthcare services. OTCAs available for purchase without prescription can also be incorporated as remedies by primary and specialist healthcare providers who are authorized to recommend elevated dosages as well. However, there is a compelling argument for establishing a general differentiation between high and low OTCA users. By categorizing individuals based on their OTCA usage patterns, the clinical relevance of the study can be significantly enhanced. The study’s measurements have focused on assessing OTCA usage in relation to stress, pain, and various facets of HRQOL.

## 5. Conclusions

This study reveals a significantly heightened risk of increased pain and stress among adolescents who are high users of over-the-counter analgesics (OTCAs) compared to their low-usage counterparts. Interestingly, our findings indicate that adolescents in the high user group exhibit a slight improvement in one aspect of HRQOL, psychological well-being, which requires further investigation to clarify the causes. Health professionals must be vigilant in identifying young individuals with high OTCA consumption and thoroughly investigate the potential connections between this medication use and their experiences of pain and stress. This awareness is crucial for developing personalized and effective interventions. Moreover, parents and caregivers play a pivotal role in guiding adolescents towards appropriate pain management strategies. They should educate their children about the indications and risks associated with OTCA use and encourage them to seek professional medical advice when necessary. Although our research provides significant insights, we acknowledge that the correlation between OTCA use and adolescents’ HRQOL is a complex issue that needs more rigorous exploration. Future studies should aim to identify the underlying mechanisms and potential moderating factors. Moreover, the findings of our study underline the importance of preventive measures. It is crucial to educate adolescents about the potential risks associated with OTCA use and to promote healthier pain management strategies. Our findings also emphasize the need for policies and interventions to reduce OTCA misuse among adolescents and ultimately improve their HRQOL.

## Figures and Tables

**Table 1:** Description of study variables.

Factors	Instruments	Number of Items
HRQOL	Kidscreen-27	
	Physical well-being	5
	Psychological well-being	7
	Autonomy and parents	7
	Social support and peers	4
	School environment	4
Pain	Brief Pain Inventory (BPI)	
	Pain on average	1
	OTCA questions	
	Intake of OTCAs during last 4 weeks	1
	Frequency of OTCA intake	1
Stress	Perceived Stress Questionnaire (PSQ)	30

**Table 2:** Characteristics of study variables.

Study Variable	Low Users (*n* = 243) (Mean/SD)	High Users (*n* = 72) (Mean/SD)
Pain intensity	2.5 (1.9)	3.4 (1.9)
Stress	0.32 (0.16)	0.38 (0.18)
HRQOL		
Physical well-being	46.25 (8.83)	43.77 (9.27)
Psychological well-being	44.90 (7.56)	43.71 (8.96)
Autonomy and parents	52.12 (7.96)	50.12 (8.53)
Social support and peers	48.15 (8.52)	46.49 (7.76)
School environment	47.14 (7.16)	44.71 (8.44)

**Table 3:** Binary multivariate logistic regressions of pain, stress, and HRQOL (independent variables) on low-(ref) and high use of OTCAs (dependent variables).

	Low (Ref) versus High OTCA Users					
	Unadjusted			Adjusted ^		
Dependent Study Variables	OR	95% CI	*p*-Value	OR	95% CI	*p*-Value
Pain	1.29	1.10 to 1.50	<0.01	1.29	1.11 to 1.51	<0.01
Stress	14.7	1.08 to 200.1	0.04	14.5	1.03 to 204.4	0.05
HRQOL:						
Physical well-being	0.99	0.95 to 1.03	0.57	0.99	0.95 to 1.03	0.48
Psychological well-being	1.08	1.02 to 1.15	0.01	1.08	1.02 to 1.15	0.02
Autonomy and parents	0.99	0.95 to 1.04	0.81	1.00	0.95 to 1.04	0.94
Social support and peers	0.98	0.94 to 1.02	0.26	0.98	0.94 to 1.02	0.29
School environment	0.98	0.93 to 1.03	0.39	0.98	0.93 to 1.03	0.49

^ Adjusted for: Gender, ethnicity, and living condition.

## Data Availability

The datasets used and/or analyzed during the present study are not publicly available due to the General Data Protection Regulation laws. However, they will be available from the corresponding author on reasonable request and with permission from the Norwegian Centre for Research Data.
